# Supplementary material for: Pathways to care for psychosis in rural Uganda: Mixed-methods study of individuals with psychosis, family members, and local leaders
Source: Glob Ment Health (Camb). 2025 Jan 3;11:e130. doi: 10.1017/gmh.2024.143 (PMC11704388; doi:10.1017/gmh.2024.143)
Supplement: Lee et al. supplementary material [file S2054425124001432sup001.docx]

**Appendix A:** Theme: Positive attitude towards biomedical providers

| **Data Extracts** |
| --- |
| "Those people can help in screening and understanding the real cause of somebody’s illness. And hence, give the required treatment. These people are educated and know what they’re doing." – IDI: Person with psychotic disorder, 43, male |
| "Hospital it worked a lot because I would get all the tabs in time and they always tell you you should do this and this in time. So I think the hospital worked a lot because they can take care of the patient." – IDI: Person with psychotic disorder, 23, male |
| "Biomedical, it works. By the time I've been coming for medication, there has been improvement. They always bring some people when they are in a bad state, but by the time we see them getting fine. Biomedical workers, they treat what they know and have seen and what you feel. And you can know when you're getting better, you can see the change." – IDI: Person with psychotic disorder, 42, male |
| "For the biomedical providers, they can scan and know the exact cause of your mental illness, then give you the exact medication for your mental illness." – IDI: Family member, 37, female |
| "None of you are more knowledgeable than the health workers, they said it was mental illness then that is it because I was there when he was diagnosed and it was only medicine that would help him" – IDI: Family member, 60, female |
| "As a parent, when you take your patient to hospital, and you see that their condition has improved. If they were not eating they can now eat, you become strong and hopeful that your patient is going to be fine." – IDI: Family member, 39, female |
| "I believe in it so much- I have too much hope that it helps get people better. I believe in those who make the medicine so they can also add knowledge to us on how to help us through the challenge. They also give information to people on how to prevent mental illness. And also giving advice." – IDI: Local leader, 49, male |
| "To give them advice and also prepare them to get help, when someone is having mental health disorder, they need to be taken to the facility for care, just like how we have right now at empower, we can advise and refer them there, we tell them that once they go there, they will find a health worker who manages mental related illnesses, he will access you and then know what you need and then you get treatment." – IDI: Local leader, 43, male |
| “Unless I have taken medicine, there is nothing that can help.” – FGD: Person with psychotic disorder |
| “The other role they [TH] have is they collaborate with religious leaders by allowing them to come pray for you at the hospitals and health facilities when you are admitted.”- FGD: Person with psychotic disorder |

**Appendix B:** Biomedical care is difficult to access

| **Data Extracts** |
| --- |
| "And then that person told me to come to the health center and then I said I could not afford transport to get to the health center." – IDI: Person with psychotic disorder, 48, male |
| "But since I’m in the village, I don’t have enough funds to buy that medication." – IDI: Person with psychotic disorder, 43, male |
| "The most effective thing could be buying him more tablets, but we can’t since we can’t afford them.” – IDI: Family member, 34, female |
| "(traditional healers) Those people have taken a lot of cash from our family. We have to bring cash and offerings, and it is a lot of money to buy their services." – IDI: Family member, 57, male |
| "Most people take so long to go and get checkups in facilities and most times some people go and seek medical care from other places instead of going to hospital to get accessed and get to know what they are suffering from" – IDI: Local leader, 43, male |
| "...health facilities are far from us. We are the last people in the village which affects us so much because you find children who have mental illness and can't know where it comes from or what has caused it." – IDI: Local leader, 41, male |
| “The only role they have is making us poor. Because there is no one who has ever gone to them and they give him a definite amount. Their things don’t end. When you become poor, that is when you will stop going there, but their things don’t end.” – FGD: Person with psychotic disorder |

**Appendix C:** Different perceived etiologies of mental illness lead individuals to different places for care

| **Perceived Cause** | **Chosen Care Provider** | **Data Extract** |
| --- | --- | --- |
| Witchcraft and/or Spirits | Traditional Healer | "They used to think I had evil spirits, or things that were attached to evil spirits."  "Some of them go to traditional healers, and they work on them and get healed." – IDI: Person with psychotic disorder, 55, male |
|  | Religious Leader | "I was unable to reproduce and my colleagues (co-wives) couldn’t wish me well. So they made witchcraft which worked upon me."   "It is the church that has enabled me to survive. Because at times, I am at church and rush to church for prayers." – IDI: Person with psychotic disorder, 70, female |
| Stress | Religious Leader | "Stress and depression."  "The best treatment was church according to my perception." – IDI: Family member, 34, female |
| Drugs/Alcohol | Biomedical | "Another thing people say, it's caused by drugs. For example, marijuana. So they believe when someone takes marijuana it brings about the issue of mental illness."  "They [biomedical providers] have the most effective way of treating mental illness because they fast test them and then find out where the problem is or what is the cause of the problem... Health personnel give advice and referrals to patients to go to another health facility or hospital" – IDI: Local leader, 49, male |
|  | Biomedical | "People could become mentally ill because they have been beaten up and sometimes sorry its because they are using drugs and substances"  "This is the best treatment since in hospitals you are screened and the exact cause of the illness is identified and in hospitals there is medicine in form of tablets and injections." – IDI: Local leader, 53, male |
| Genetics | Biomedical | "If a family also has history of madness, it might also look for someone else, following someone."  "I have been encouraging them to go to hospitals, to meet a psychiatric Doctor." – IDI: Local leader, 33, male |
| Fever or Previous Illness/Injury | Biomedical | "If you get a fever and you fail to go to the hospital the fever can easily interfere with your reasoning and mental ability this implies that someone is becoming mentally ill just because they failed to go to the hospital"  "Or medication, through the health centers is very good and I would recommend that since in those places, people are screened and the exact cause of the illnesses are always found out so they treat what they understand, and people always get better." – IDI: Local leader, 37, male |
